# Occurrence of *Listeria monocytogenes* in Raw Bovine Milk from Ecuador: Culture-Based Isolation and Molecular Identification

**DOI:** 10.3390/vetsci13090871

**Published:** 2026-08-27

**Authors:** Byron Puga-Torres, Anthony Loor-Giler, Camila Sánchez-Castro, Andrea Padilla-Cerda, Silvana Santander-Parra, Luis Núñez

**Affiliations:** 1 Facultad de Medicina Veterinaria y Zootecnia, Universidad Central del Ecuador, Jerónimo Leyton S/N y Gilberto Gatto Sobral, Quito EC 170521, Ecuador; bpuga@uce.edu.ec; 2 Laboratorios de Investigación, Dirección General de Investigación, Universidad de las Américas (UDLA), Antigua Vía a Nayón S/N, Quito EC 170124, Ecuador; a.abel.loor.giler@gmail.com (A.L.-G.); andrea.padilla.cerda@outlook.com (A.P.-C.); 3 Facultad de Ciencias Veterinarias, Universidad de Buenos Aires, Av. Chorroarín 280, Buenos Aires 1427, Argentina; 4 Facultad de Ingeniería y Ciencias Aplicadas, Carrera de Ingeniería en Biotecnología, Universidad de Las Américas (UDLA), Antigua Vía a Nayón S/N, Quito EC 170124, Ecuador; camila.sanchez.castro285@hotmail.com; 5 Facultad de Ciencias de la Salud, Carrera de Medicina Veterinaria, Universidad de Las Américas, Antigua Vía a Nayón S/N, Quito EC 170124, Ecuador; silvanahsp@yahoo.com; 6 One Health Research Group, Facultad de Ciencias de la Salud, Universidad de Las Americas, Quito EC 170124, Ecuador

**Keywords:** raw milk, *Listeria monocytogenes*, Ecuador, Manabí, Pichincha, prevalence, qPCR

## Abstract

**Simple Summary:**

*Listeria monocytogenes* is a foodborne bacterium that can contaminate raw milk and dairy products. This study evaluated the occurrence of *L. monocytogenes* in raw bovine milk collected from dairy farms in two provinces of Ecuador using molecular and culture-based detection methods. A total of 633 milk samples were analyzed, and 27.33% tested positive for the bacterium. Significant differences were observed between provinces and climatic seasons, with higher positivity detected in Pichincha and during the rainy season. qPCR showed high relative sensitivity and specificity compared with Bacterial culture, supporting its usefulness as a rapid screening method. The findings of this study demonstrate the presence of *L. monocytogenes* in raw milk from the regions under investigation. This finding serves to emphasise the significance of adhering to stringent hygienic practices and conducting rigorous microbiological monitoring during the processes of milk production and handling.

**Abstract:**

*Listeria monocytogenes* is one of the main foodborne pathogens associated with milk and dairy products due to its ability to survive under refrigeration conditions and persist in dairy production environments. The aim of this study was to determine the occurrence of *L. monocytogenes* in raw bovine milk samples collected from dairy farms in the provinces of Pichincha and Manabí, Ecuador, using culture-based and molecular methods. A total of 633 raw milk samples were collected between September 2022 and July 2023 from small-, medium-, and large-scale producers. Detection was performed by qPCR targeting the *hly* gene after pre-enrichment, followed by Bacterial culture and molecular confirmation through *16S rRNA* gene analysis. Overall, 27.33% (173/633) of the samples tested positive for *L. monocytogenes*. Positive samples were distributed among small-scale producers (35.84%), medium-scale producers (36.99%), and large-scale producers (27.17%). Statistically significant differences were observed between provinces (*p* < 0.0001). Multivariable logistic regression analysis showed that samples obtained in Pichincha had considerably greater probabilities of being positive compared to those from Manabí (OR = 2.37; 95% CI: 1.61–3.48; *p* < 0.001). However, during the dry season, samples collected in Manabi showed significant probabilities of being positive compared to the rainy season (OR = 0.31; 95% CI: 0.21–0.45; *p* < 0.001). Producer size was not significantly associated with positivity after adjustment. Agreement between qPCR and culture-based methods was moderate (κ ≈ 0.6). Relative diagnostic performance analysis demonstrated that qPCR showed a relative sensitivity of 92.35%, relative specificity of 96.54%, positive predictive value (PPV) of 90.75%, and negative predictive value (NPV) of 97.17% compared with Bacterial culture. These findings confirm the occurrence of *L. monocytogenes* in raw bovine milk from the studied regions and highlight the importance of maintaining hygienic practices and microbiological surveillance during milk production and handling.

## 1. Introduction

Bovine milk is one of the most complete foods found in nature due to its huge nutritional value, which provides a combination of carbohydrates, lipids, proteins, vitamins and minerals that are essential for human nutrition [1]. However, because of these nutritional qualities, raw milk becomes the perfect culture media for bacterial growth. It also contains a lot of water, has a pH that is almost neutral (between 6.6 and 6.8), and is secreted at the cow’s body temperature, which is between 37 and 38 °C. Endogenous (diseases of the animal or the mammary gland) or exogenous (the entire environment surrounding dairy production, including personnel, ambient conditions, environment, environmental hygiene, milking, transportation, and storage, among others) contamination can come from a variety of sources [2,3,4,5].

In Ecuador, in 2024, an approximate daily production of 5.3 million liters of milk was reported; 79.5% was produced in the inter-Andean region, 16.3% on the coast, and 4.2% in the Amazon region. The inter-Andean province of Pichincha generates 758,503 L of milk daily, accounting for 13.59% of Ecuador’s total production. In contrast, the coastal province of Manabí produces 631,022 L per day, representing 11.31% of the national output. Collectively, these provinces contribute approximately 25% of Ecuador’s milk supply [6]. The majority of dairy cows come from small (between 1 and 20 cows in production) and medium (between 21 and 100 lactating animals) producers with little technological or productive efficiency. These producers represent nearly 80% of the total milk produced in the country, and are crucial to its economy, generating nearly 1.5 million jobs annually [7].

Ecuador is self-sufficient in dairy production; however, only a little over 15% of production reaches the industry. Therefore, the remaining volume is marketed informally (either in liquid form or transformed into unpasteurized dairy products). This constitutes one of the greatest risks, as the safety of the products is not guaranteed [7,8,9]. Of all the risks that raw milk consumption can pose, the presence of antibiotic residues along with pathogenic and zoonotic microorganisms, such as *L. monocytogenes*, is the greatest concern for public health [8]. Thus, several studies carried out on raw milk from Ecuador, especially on milk marketed informally, have confirmed serious non-compliance with the Ecuadorian Technical Standard (NTE) INEN 9, with a high presence of antibiotic residues and various pathogenic bacteria such as *Escherichia coli* O157:H7, *Salmonella* spp., *Campylobacter coli*, *Campylobacter jejuni* and *Staphylococcus aureus* [10,11,12,13,14,15,16,17].

*L. monocytogenes* is a Gram-positive, facultative anaerobic, catalase-positive, non-encapsulated and non-sporulated bacillus capable of adapting to different environments by resisting a pH between 4.39 and 9.40. It possesses flagella, and its motility is temperature-dependent, being typically expressed at lower temperatures. Additionally, it can develop increased resistance to disinfectants through the formation of biofilms, which enhance its persistence on surfaces and in food-processing environments [18,19]. It belongs to the *Listeriaceae* family and is made up of 29 species [20]; however, it is the main cause of listeriosis: *L. ivanovii* in ruminants and *L. monocytogenes* in humans. *L. monocytogenes* is classified into 13 serotypes: 1/2a, 1/2b, 1/2c, 3a, 3b, 3c, 4a, 4ab, 4b, 4c, 4d, 4e, and 7. They are designated by the use of an immunoreactive reagent for the H and O antigens found on surface cells [21,22].

The consumption of food contaminated with these bacteria can lead to listeriosis, causing meningitis, encephalitis, and septicemia; while in animals it causes encephalitis, abortion, and septicemia [21,23,24]. Following the ingestion of contaminated food, listeriosis may initially present with mild, flu-like symptoms such as fever, muscle aches, nausea, or diarrhea, which can progress to severe invasive disease when the bacteria cross the intestinal barrier and disseminate systemically [25]. The disease is aggravated in the most vulnerable groups, such as pregnant women (causing abortions or premature births), the elderly, newborns, and immunosuppressed individuals [21,23,24].

Although listeriosis is a well-recognized foodborne disease linked to eating tainted dairy products, there are currently few or poorly documented cases in Ecuador that are directly related to consuming raw cow’s milk in the scientific literature. However, international evidence has clearly demonstrated outbreaks associated with the consumption of unpasteurized milk and dairy products. Thus, this is then considered a high route and a significant risk factor for transmission. For instance, outbreaks in the United States and Europe have been linked to raw milk consumption, where *L. monocytogenes* was isolated from both patients and implicated dairy products [26,27]. These findings underscore the public health importance of controlling raw milk consumption, particularly in regions like Ecuador where informal milk markets are prevalent [15,16,17,28].

In individuals over 60 years of age, listeriosis causes the highest mortality rate, reaching 74% of cases, while children have the lowest rate at 1% [29]. In Mali, the morbidity rate from perinatal listeriosis was 21%, with newborns presenting with respiratory symptoms, meningitis, and in many cases, death. There are even reports of the presence of *L. monocytogenes* in breast milk, so an infected breastfeeding mother can transmit the bacteria to her children [30].

For this reason, the presence of *L. monocytogenes* in food is mandatory for reporting in industrialized countries. This is because it can grow in contaminated water, making it an ideal medium for spreading, as demonstrated by reports from countries in the United States [31]. Another source of contamination is raw meat products, as well as unpasteurized milk and dairy products made from it. In the latter two cases, despite storing a dairy product at 6–7 °C, the presence of *L. monocytogenes* can reach counts higher than 10^4^ CFU/g [22,24].

In addition, ready-to-eat (RTE) foods are considered one of the most important vehicles for the transmission of *L. monocytogenes*, as they are consumed without further heat treatment, allowing the pathogen to persist and multiply during storage. High-risk RTE foods include soft cheeses, deli meats, smoked fish, and pre-packaged salads, which have been frequently associated with listeriosis outbreaks worldwide. The ability of *L. monocytogenes* to grow at refrigeration temperatures and form biofilms on food-contact surfaces further increases the risk of contamination in these products, making them a major concern for food safety and public health [32,33,34].

Considering that in Ecuador a significant proportion of milk and dairy products are obtained from the informal market, artisanal processing is carried out without the implementation of quality control measures to ensure product safety. In addition, *L. monocytogenes* has been identified as one of the most serious pathogens with the potential to affect public health. This research is of particular importance because it addresses a critical gap in food safety surveillance in regions where raw milk consumption is common and regulatory control is limited. Furthermore, the identification of the prevalence of this pathogen provides essential baseline data for risk assessment, supporting the development of targeted public health interventions, contributing to the implementation of policies intended to lower the risk of listeriosis transmission through dairy products, particularly among vulnerable populations. The objective of this research was to determine the prevalence of these bacteria in raw milk from the provinces of Pichincha and Manabí, the largest producers in the inter-Andean region and the Ecuadorian coast, respectively.

## 2. Materials and Methods

### 2.1. Population and Samples

**Figure 1:**
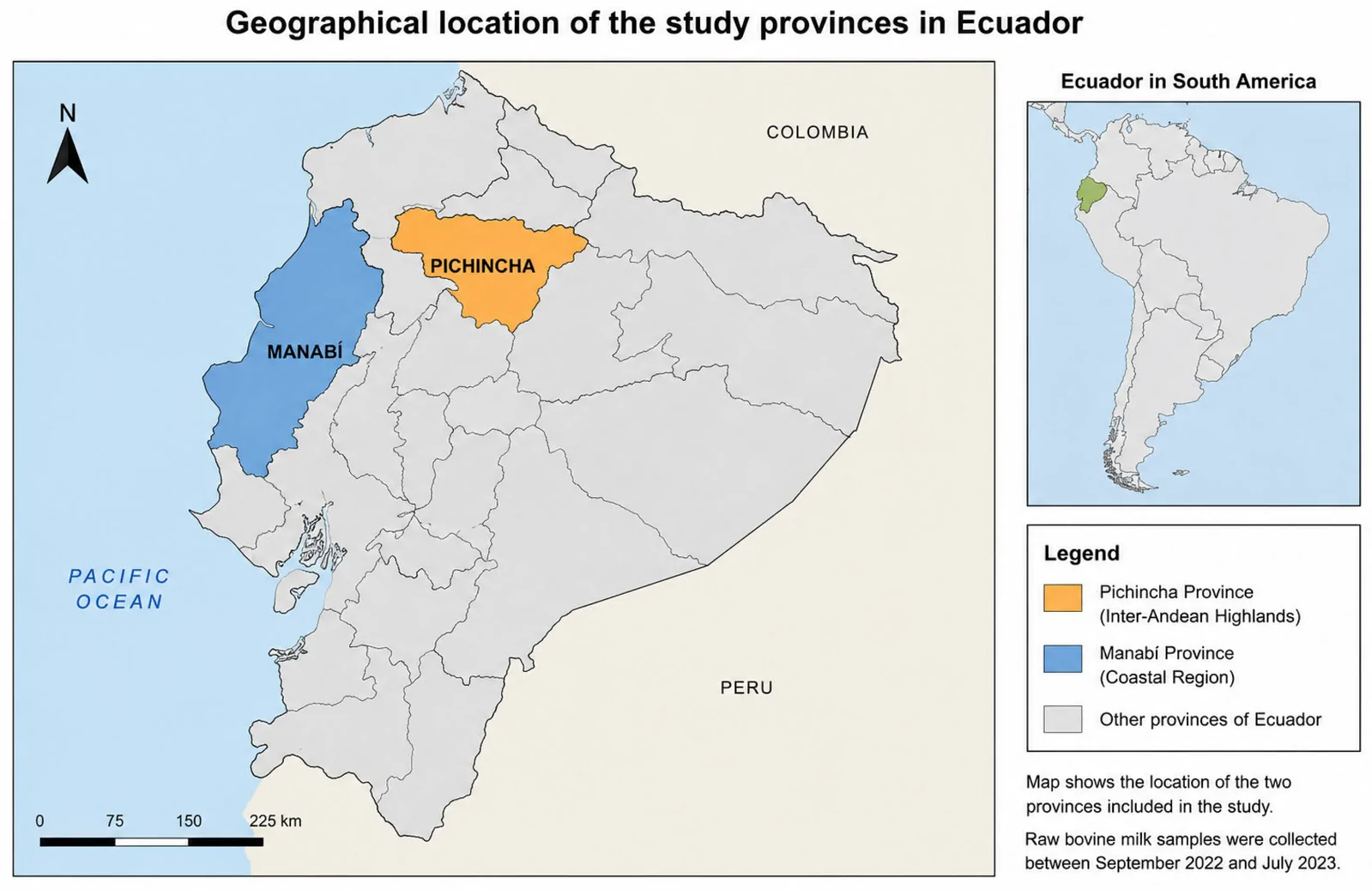
Geographical location of the provinces included in the study. The map shows the location of Pichincha Province (Inter-Andean Highlands) and Manabí Province (Coastal Region), Ecuador.

The samples (100 mL) were collected in sterile containers by personnel from the Universidad Central del Ecuador directly from the bulk milk cooling tanks of dairy producers in the provinces of Pichincha and Manabí, Ecuador, between September 2022 and July 2023 (Figure 1), following the guidelines established in NTE INEN ISO 707 [35]. Dairy farms were classified according to herd size as small (1–20 cows in production), medium (21–100 cows), and large (>100 cows in production). A non-probability convenience sampling strategy was employed. Dairy farms were invited to participate voluntarily in the study, and only those producers who agreed to participate and met the inclusion criteria were sampled. Therefore, the number of farms included in each producer category was determined by producer participation during the study period rather than by a predefined proportional or stratified sampling design. Consequently, the final number of samples within each producer category, province, and climatic season was not identical, reflecting field conditions and voluntary participation rather than a predefined balanced sampling design. The producer classification was used to characterize the study population and to evaluate its potential association with the occurrence of *L. monocytogenes*, but not as a sampling stratum. At the end of the sampling phase, 633 raw milk samples were taken, 322 from the province of Pichincha and 311 from Manabí [Supplementary Material Data S1].

Sampling was conducted during both the dry and rainy seasons to include the climatic variability of the study area; however, farms were enrolled according to producer availability and willingness to participate rather than through random selection.

Samples considered “dry” are those collected between June and September, a climatic period characterized by drought and high temperatures (≈30 °C), while those considered “rainy” are those collected between October and May, a period characterized by abundant rainfall and low temperatures (≈22 °C). These data are stipulated according to the National Institute of Meteorology and Hydrology (INAMHI). All samples were collected from different producers; no sample was collected from different containers of the same producer, whether small, medium, or large. The samples were stored in coolers with cooling gels, maintaining the cold chain (2–6 °C) from the collection and during transport to the Laboratory of the Universidad de Las Americas (UDLA), where bacteriological and molecular analysis was carried out.

Although all samples were obtained from different producers and only one bulk tank sample was collected from each participating farm, farms located within the same province may share environmental, climatic, or management characteristics that were not directly measured in this study. These shared characteristics could act as unmeasured confounding factors influencing the occurrence of *L. monocytogenes*. However, because each farm contributed only one independent observation, no within-farm clustering structure was present that would require cluster-based statistical correction.

Furthermore, because the study used convenience sampling rather than probability-based sampling, caution should be exercised when extrapolating prevalence estimates to the entire dairy-producing population of Ecuador. To reduce this potential bias, samples were distributed among different producer categories (small, medium, and large farms) and collected across two geographically distinct provinces and different climatic periods. Nevertheless, the results should be interpreted cautiously, particularly regarding prevalence extrapolation and statistical inference at the population level.

All procedures performed in this study were approved by the Committee for the Care and Use of Domestic and Laboratory Animal Resources of the Agency for Regulation and Phytosanitary and Zoosanitary Control of Ecuador (AGROCALIDAD) under number INT/DA/019 (Approved: 31 January 2018).

#### Sample Size Calculation

The number of samples needed for this study was determined based on an expected prevalence of *L. monocytogenes* of 50%, a desired absolute precision of 5%, and a 95% confidence level. The sample size was determined using the standard formula:

$$n=\frac{Z^{2}*P_{exp}(1-P_{exp})}{d^{2}}$$

where *n* is the required sample size, *Pexp* is the expected prevalence (0.50), *d* is the desired absolute precision (0.05), and *Z* is the statistic for a 95% confidence level (1.96). Based on this formula, the minimum number of raw milk samples required to estimate the prevalence of *L. monocytogenes* was calculated to be 384 samples.

### 2.2. Enrichment of Raw Milk Samples

The 633 collected samples were enriched in Brain Heart Infusion Broth (BHI) (Difco™, Franklin Lakes, NJ, USA, CAT 237500), in a ratio of 90% liquid medium to 10% sample (raw bovine milk). The enrichments were incubated at 37 °C for ~19 h with constant shaking at 150 rpm.

### 2.3. DNA Extraction from Enriched Milk

A 1 mL aliquot of enriched milk was taken, centrifuged at 12,000× *g* for 15 min, the supernatant was discarded and resuspended in 200 µL of TE Buffer (Invitrogen™, Carlsbad, CA 92008, USA). The result was subjected to bacterial DNA extraction using the Phenol/chloroform-based method with GT reagent according to the previously described protocol [36]. The extracted DNA was stored at −20 °C until use for qPCR identification of *L. monocytogenes*.

### 2.4. Bacterial Culture Using Selective Media

After incubation in BHI, samples were plated on Modified Chromogenic Listeria Agar (TM MEDIA, Delhi, India, CAT TM 1634), a selective chromogenic medium commonly used for the presumptive isolation of *L. monocytogenes*. Although this plating step was inspired by selective culture approaches described in ISO 11290-1:2004 [37], the overall methodology used in this study did not follow the complete ISO 11290-1 workflow. The plates were incubated at 37 °C for at least 24 h, after which blue and halo colonies were identified as positive for *Listeria* spp. This approach does not strictly follow ISO 11290-1:2017 but was complemented with molecular confirmation to ensure specificity. *L. monocytogenes* ATCC 13932 was also subjected to Bacterial culture procedures described here.

### 2.5. DNA Extraction from Bacterial Isolates

Three to six colonies characteristic of *Listeria* spp. were collected and placed in 1.5 mL Eppendorf tubes containing 200 µL of TE Buffer (Invitrogen™, Carlsbad, CA 92008, USA), for DNA extraction; the mixture was vortexed and placed at −80 °C for at least 10 min. Samples were then heated on a heat block at 95 °C for 1 min, vortexed again, and centrifuged at 12,000× *g* for 10 min at 4 °C [38]. After centrifugation, 100 µL of the supernatant was placed in new tubes and stored at −20 °C until use for identification and confirmation by qPCR that the colony phenotypically characterized as *Listeria* spp. is indeed *L. monocytogenes*. DNA of *L. monocytogenes* ATCC 13932 was also extracted as described here.

### 2.6. Real-Time PCR for the Confirmation and Identification of L. monocytogenes

A real-time PCR reaction was performed based on primers and hydrolysis probes (Table 1), oriented to amplify a specific region of *hly* gene to avoid the amplification of another serovar of *Listeria*. In order to determination of sensitivity of assay the construction of the standard curve was carried out [Supplementary Material Data S2], so the target fragment was amplified in an end-point PCR using only the primers hlyQF and hlyQR (Table 1) using the Platinum Taq DNA polymerase (Thermo Fisher Scientific, Carlsbad, CA, USA) according to the manufacturer’s instructions, using 1 U of Taq Polymerase and 50 mM of magnesium chloride. After amplicon confirmation, DNA was subjected to enzyme purification with ExoZap-IT kit (Applied Biosystems by Thermo Fisher Scientific, Santa Clara, CA 95051, USA), and was used as the basis for the standard curve construction. Using the web tool DNA copy number and dilution Calculator (Thermo Fisher Scientific) was determined the quantity of DNA necessary to make a first dilution with a known quantity of DNA copies (10^9^ copies), then serial dilutions in base 10 were performed, resulting in a standard curve from 10^9^ copies to 1 DNA copy numbers. The qPCR reaction contained 2X TaqMan™ Universal Master Mix II, with UNG (Applied Biosystems, Waltham, MA, USA), 0.2 µM of each primer, 0.1 µM of hydrolysis probe (Table 1), 1 µL of DNA extracted from pre-enriched milk or bacterial isolates and the volume of 10 µL was completed with UltraPure™ DNase/RNase-Free Distilled Water (Thermo Fisher Scientific, USA, CAT 10977015). The reactions were executed in a CFX96 Touch Real-Time PCR Detection System thermal cycler (Bio-Rad Laboratories, Inc., Hercules, CA 94547, USA) following the thermal cycler conditions previously described [39]. Each sample, including DNA extracted from isolated *Listeria* spp. colonies and DNA extracted from enriched milk as described above, was analyzed in duplicate. A positive control (*L. monocytogenes* ATCC 13932), a non-template control (ddH_2_O), and appropriate negative controls were included in each qPCR run to verify assay performance. All qPCR-positive isolates were subsequently re-cultured on selective media, confirmed for purity, and stored at –80 °C for further analyses.

### 2.7. Estimated L. monocytogenes Gene Copy Number in Enriched Raw Milk

Absolute quantification of post-enrichment *L. monocytogenes* gene copy number (gene copies/µL) was calculated from the qPCR standard curve results [Supplementary Material Data S1]. One microliter of extracted DNA was used as template in the qPCR reaction. Prior to amplification, DNA extracts were diluted 1:5 to reduce the potential effects of PCR inhibitors commonly associated with milk-derived DNA extracts. However, an internal amplification control was not included; therefore, residual inhibition was not quantitatively assessed for individual samples. Because DNA extraction was performed after the selective enrichment step, the estimated gene copy numbers represent the amount of bacterial DNA detected after enrichment and should not be interpreted as the original concentration of *L. monocytogenes* in the raw milk samples.

DNA was extracted from 1 mL of pre-enrichment culture as described above, and the final DNA extract volume obtained was 30 μL. Consequently, the estimated number of gene copies per microliter of extracted DNA was multiplied by 30 to obtain the total number of gene copies recovered from the 1 mL pre-enrichment sample. Because the pre-enrichment step was performed using a 1:10 dilution of raw milk in BHI broth, the resulting value was further multiplied by 10 to estimate the number of genome equivalents per milliliter of raw milk.

The final calculation was performed as follows: Estimated post-enrichment genome equivalents recovered from enriched milk = (gene copies quantified by qPCR × dilution factor × final DNA volume) × pre-enrichment correction factor. Where the dilution factor was 5, the final DNA extraction volume was 30 μL, and the pre-enrichment correction factor was 10.

Thus: Estimated post-enrichment gene copy number recovered after enrichment = qPCR gene copies × 5 × 30 × 10.

### 2.8. End-Point PCR and 16S Gene Sequencing

Five DNA samples were randomly taken from isolated bacteria (*L. Monocytogenes),* and end-point PCR was performed for the amplification of the *16S* ribosomal gene using the previously described primers 27F and 1482R (Table 1) to analyze the characteristics of the *16S* gene present in isolated strains of *L. monocytogenes*. PCR protocols were executed using the PlatinumTM Taq DNA Polymerase Kit (Invitrogen by Thermo Fisher Scientific, Carlsbad, CAT 10966018, USA) using 1 U of Taq Polymerase, 0.5 µM of each primer (27F and 1482) and 50 mM of magnesium chloride. After thermocycling, the PCR products were analyzed on a 2% agarose gel stained with SYBR Safe DNA Gel Stain (Invitrogen by Thermo Fisher Scientific, Carlsbad, CA S33102, USA). The visualized amplified samples were purified with the ExoSAP-IT™ Express PCR product Cleanup Kit (Applied Biosystems, Santa Clara, CA 95051, USA) according to the manufacturer’s instructions using 2 µL of ExoSAP-IT™ Express reagent for every 5 µL of each post-PCR reaction product. The purified samples were sequenced using the Big Dye Terminator v 3.1 cyclic sequencing kit (Applied Biosystems by Thermo Fisher Scientific, Foster City, CA 94404, USA) and analyzed using the ABI 3500 DNA analyzer (Applied Biosystems, Thermo Fisher Scientific). The resulting electropherograms were curated using the Geneious prime version 2024.0.7 program (https://www.geneious.com) and the obtained sequences were aligned with sequences reported in GenBank of different *Listeria* species randomly selected using the CLUSTAL X 2.0.11 software [41], and a nucleotide similarity matrix and a phylogenetic tree were constructed. The phylogenetic tree was inferred using the neighbor-joining statistics method with a p-distance substitution model and a bootstrap of 1000 replicates through the MEGA version 7 software [42].

### 2.9. Statistical Analysis

Descriptive statistics were used to measure the prevalence (number of positive samples/number of total samples) of *L. monocytogenes* in raw milk samples from the provinces of Pichincha and Manabí. The results were then compared between provinces and producer types using the chi-square test. Prevalence estimates were additionally expressed with 95% confidence intervals (95% CI). Furthermore, univariable and multivariable logistic regression analyses were performed to evaluate the association between *L. monocytogenes* positivity and explanatory variables, including province, producer type, and climatic season. Because the study employed a non-probability convenience sampling design, the distribution of samples among provinces, producer categories, and climatic seasons was not predetermined or balanced. Therefore, these variables were included in the regression models to account for their potential association with *L. monocytogenes* occurrence despite the unequal sample distribution. Odd ratios (ORs) were reported together with their corresponding 95% confidence intervals (95% CI). To determine the difference between the pre-enriched milk qPCR and Bacterial culture assays, the level of agreement between the *L. monocytogenes* detection results of these two methods was evaluated using Cohen’s kappa coefficient. Additionally, the diagnostic performance of qPCR relative to Bacterial culture was assessed by calculating sensitivity, specificity, positive predictive value (PPV), and negative predictive value (NPV). To further evaluate the diagnostic performance of qPCR relative to Bacterial culture, a two-by-two contingency table was constructed using Bacterial culture as the reference method. Relative sensitivity, relative specificity, positive predictive value (PPV), and negative predictive value (NPV) were calculated according to the recommendations of the World Organization for Animal Health (WOAH) for diagnostic test evaluation and validation [43]. Relative sensitivity was calculated as TP/(TP + FN), relative specificity as TN/(TN + FP), PPV as TP/(TP + FP), and NPV as TN/(TN + FN), where TP represents true positives, TN true negatives, FP false positives, and FN false negatives. Because Bacterial culture is not considered a perfect gold standard for the detection of *L. monocytogenes*, these parameters are reported as relative measures of diagnostic performance. Given the observational nature of the study and the convenience sampling strategy, statistical analyses were intended to evaluate associations within the sampled population rather than to provide population-level estimates representative of all dairy farms in Ecuador.

Therefore, the calculated sensitivity, specificity, PPV, and NPV are presented as relative diagnostic performance measures and should not be interpreted as absolute estimates of qPCR accuracy or bacterial viability.

For this purpose, the free statistical software “R Studio” version 1.2.5019 (RStudio Inc., Boston, MA, USA) was used. The confidence level for these tests was 95%.

### 2.10. Sequences Accession Numbers

The nucleotide sequences generated in this study were deposited in GenBank under accession numbers PZ207425–PZ207429, corresponding to samples 3LT_ECU_UDLA, 205LT_ECU_UDLA, 127LT_ECU_UDLA, 502LT_ECU_UDLA, and 2LT_ECU_UDLA, respectively.

## 3. Results

### 3.1. Isolation of L. monocytogenes from Raw Milk

Colonies exhibiting a smooth greenish appearance surrounded by a black halo on a light background were considered presumptive *L. monocytogenes* and were phenotypically characterized on the selective medium. Presumptive isolates were subsequently confirmed by qPCR through the amplification of a species-specific gene, as described in the Materials and Methods section. Among the 633 raw milk samples collected from the provinces of Pichincha and Manabí and subjected to Bacterial culture, *L. monocytogenes* was confirmed in 170 samples, corresponding to an overall prevalence of 26.86% (170/633), whereas the remaining 463 samples (73.14%) were negative for *L. monocytogenes* (Table 2).

A multivariable logistic regression model was performed to evaluate the association between *L. monocytogenes* positivity and selected explanatory variables. After adjustment, samples collected in Pichincha province showed significantly higher odds of positivity compared with Manabí province (OR = 2.37; 95% CI: 1.61–3.48; *p* < 0.001). Likewise, samples collected during the dry season showed significantly lower odds of positivity compared with the rainy season (OR = 0.31; 95% CI: 0.21–0.45; *p* < 0.001). Producer size was not significantly associated with positivity after adjustment (*p* > 0.05), as described in Table 3.

Although producer category showed small numerical differences in the crude prevalence estimates, neither the univariable nor the multivariable logistic regression analyses identified producer category as a significant predictor of *L. monocytogenes* positivity. After adjustment for province and climatic season, the observed differences remained non-significant, indicating that producer category was not an independent risk factor.

### 3.2. Molecular Diagnosis of L. monocytogenes in Raw Milk

The qPCR test described previously [29], showed a sensibility of one copy of genetic material with a standard curve with 97.7% efficiency and a correlation coefficient of 0.998 [Supplementary Material Data S2]. Samples with a Ct of less than 38 (equivalent to one copy) were considered positive.

Regarding the detection of *L. monocytogenes* by post-enrichment qPCR, molecular analysis of the 633 raw bovine milk samples collected from the provinces of Pichincha and Manabí (Ecuador) identified *L. monocytogenes* in 173 samples, corresponding to an overall prevalence of 27.33% (173/633), whereas the remaining 460 samples (72.67%) tested negative (Table 4).

**Figure 2:**
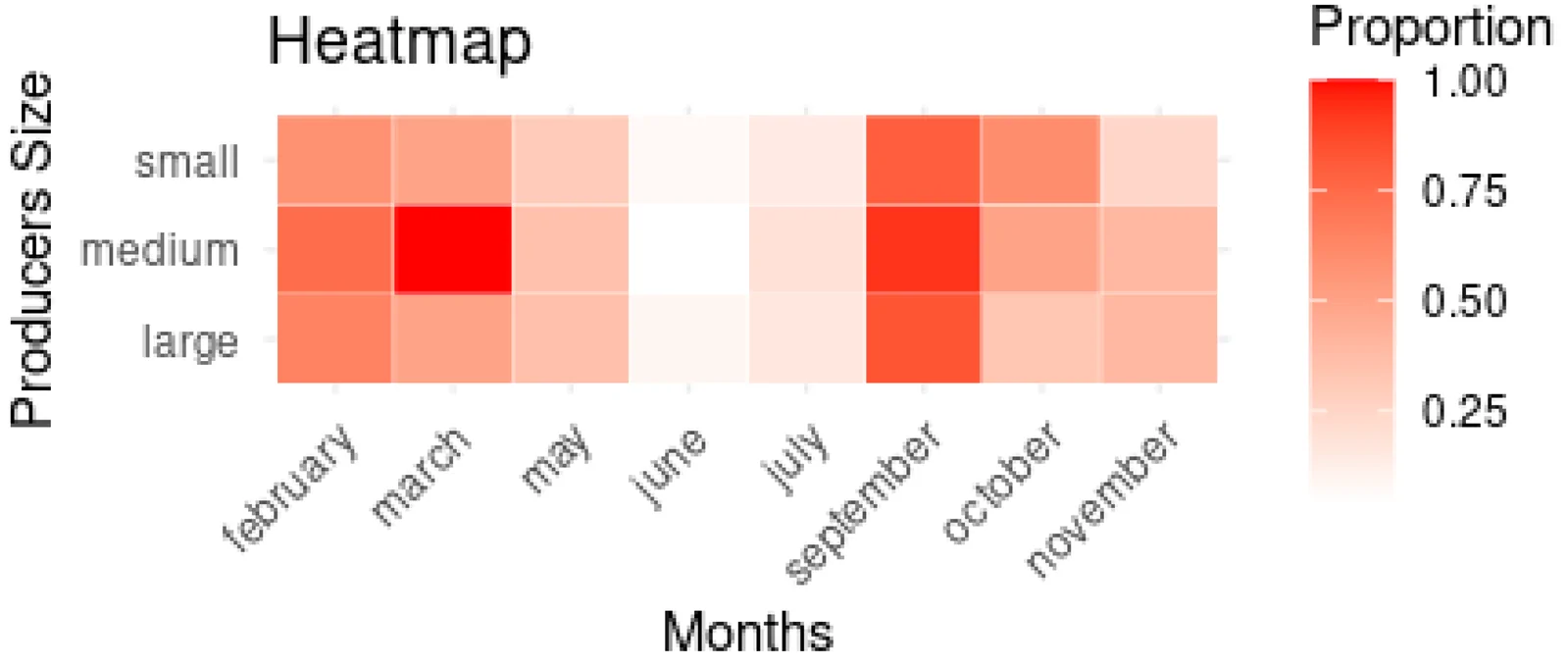
Illustrative heat map of the distribution of *L. monocytogenes* based on the month of sample collection (between February and November) and the size of the producer (small, medium and large) that supplied the sample, according to qPCR in enrichment milk data. A color scale on the right indicates proportion levels from zero (light) to one (dark red).

Of the positive samples, 35.84% (62/173) originated from small-scale producers, 36.99% (64/173) from medium-scale producers, and 27.17% (47/173) from large-scale producers (Table 4). No significant differences were found among producer categories (*p* = 0.3612). Likewise, no significant differences were observed between the dry and rainy seasons regarding the presence of *L. monocytogenes*. Nevertheless, a temporal variation in the proportion of positive samples was observed throughout the sampling period, with higher proportions detected in September, February, and March, reaching the highest monthly prevalence in mid-March (Figure 2).

These results demonstrate a consistent detection of the pathogen across both methods, although the agreement between them was moderate (κ ≈ 0.6). This level of agreement suggests that qPCR and culture-based methods may detect different fractions of the bacterial population, as qPCR can amplify DNA from both viable and non-viable cells, whereas Bacterial culture confirms only viable organisms.

Relative diagnostic performance analysis showed that qPCR detection in pre-enriched milk exhibited a relative sensitivity of 92.35% (95% CI), relative specificity of 96.54% (95% CI), positive predictive value (PPV) of 90.75%, and negative predictive value (NPV) of 97.17% when compared with Bacterial culture. These findings support the use of qPCR as a rapid and sensitive screening tool for the detection of *L. monocytogenes* in raw milk samples, while culture-based isolation remains necessary to confirm bacterial viability.

Therefore, the combined use of both methods provides a more comprehensive detection approach, and although enumeration was not performed, the comparable prevalence obtained by both methods suggests that enrichment did not significantly affect detection outcomes.

### 3.3. Estimated L. monocytogenes Gene Copy Number in Raw Milk

Absolute quantification analysis based on the qPCR standard curve indicated the presence of *L. monocytogenes* DNA in a subset of positive samples. Among the 173 qPCR-positive samples, the estimated post-enrichment *L. monocytogenes* gene load showed a median of 4.19 × 10^4^ gene copies/mL, with a first quartile (Q1) of 1.93 × 10^4^ gene copies/mL and a third quartile (Q3) of 1.02 × 10^5^ gene copies/mL. The interquartile range (IQR) was 8.25 × 10^4^ gene copies/mL. Values ranged from 1.67 × 10^3^ to 3.97 × 10^14^ gene copies/mL. Because DNA extraction was performed after selective enrichment, the estimated gene copy numbers represent post-enrichment molecular measurements rather than the original bacterial concentration present in raw milk. Therefore, these values should be interpreted only as an estimate of bacterial DNA recovered after enrichment and not as a direct quantification of viable bacteria in the original samples.

### 3.4. Analysis of 16S Gene Sequences

**Figure 3:**
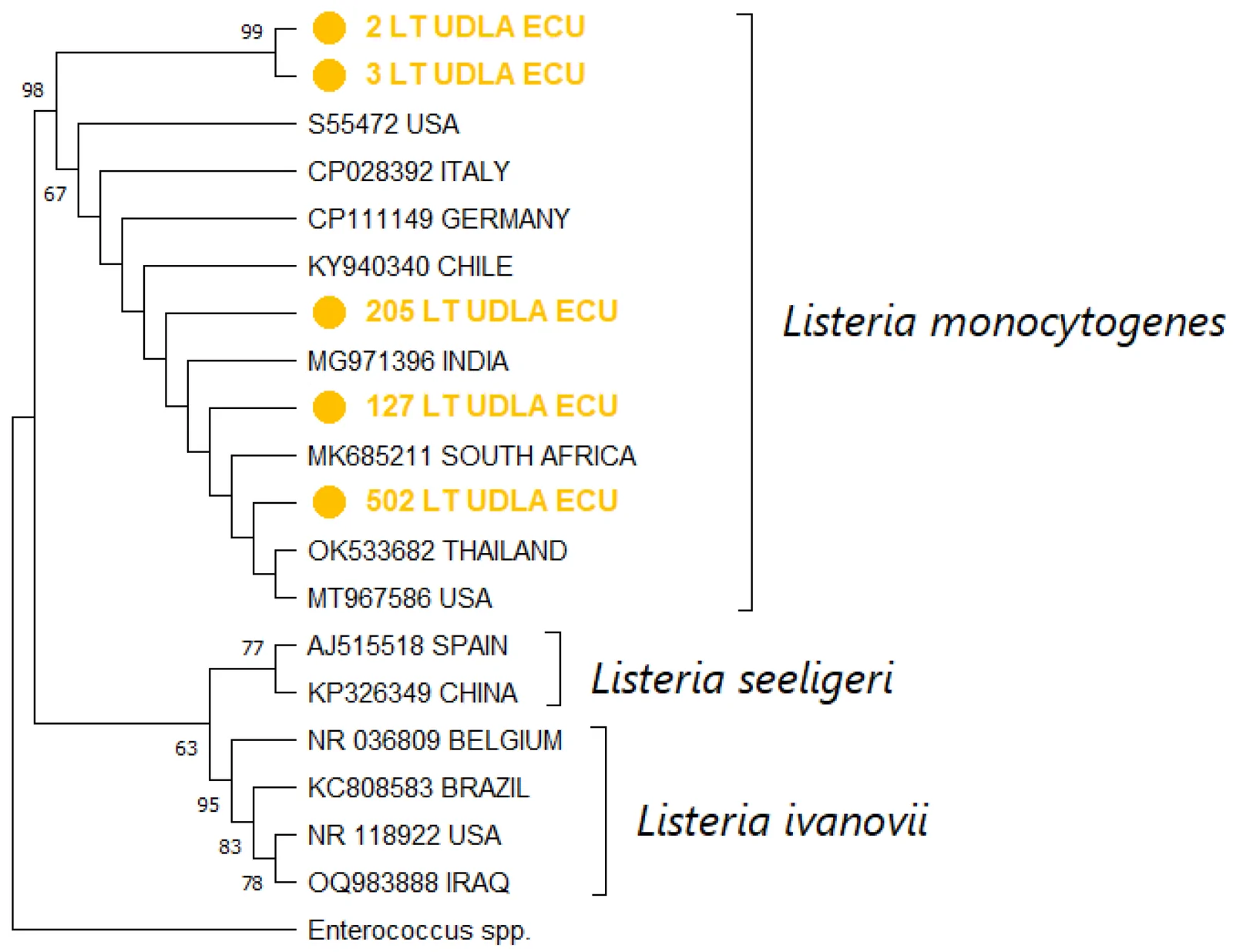
Phylogenetic relationships between *L. seeligeri*, *L. ivanovii*, and *L. monocytogenes* sequences obtained in this study and sequences from other countries. The sequences were aligned using the CLUSTAL W method in ClustalX2 2.1. The phylogenetic tree was constructed using the MEGA 7 software package. The numbers along the branches refer to bootstrap values for 1000 replicates. The scale bar represents the number of substitutions per site. A 16S sequence of *Enterococcus* spp. was used as the outgroup. The sequences obtained in this study appear in yellow and are marked with a yellow circle “•”.

Analysis of the *16S rRNA* gene sequences revealed a high degree of nucleotide similarity with reference sequences of *Listeria* spp., further supporting the qPCR results. Sequencing of the five randomly selected isolates showed 95–100% nucleotide similarity among the study sequences and 98–100% similarity with *L. monocytogenes* reference sequences deposited in GenBank [Supplementary Material Data S3]. These results provided additional taxonomic confirmation of the five sequenced isolates but were not intended to confirm or represent the complete collection of 170 isolates. Phylogenetic analysis showed that the sequences generated in this study clustered within a well-supported clade (bootstrap = 98%) together with *L. monocytogenes* reference sequences from GenBank, clearly separated from the clades corresponding to *L. seeligeri* and *L. ivanovii* (Figure 3).

The raw data used to calculate the diagnostic performance parameters are presented in Table 5, showing 157 true positives, 447 true negatives, 16 false positives, and 13 false negatives obtained by comparing post-enrichment qPCR with Bacterial culture.

## 4. Discussion

The present study demonstrates that combining pre-enrichment qPCR with conventional Bacterial culture provides a robust approach for detecting *L. monocytogenes* in raw bovine milk. The qPCR assay targeted the *hly* gene, a species-specific marker that enables reliable detection of *L. monocytogenes* without cross-reactivity with other *Listeria* species [39]. While qPCR offers rapid and sensitive detection of bacterial DNA, culture-based isolation remains essential to confirm bacterial viability. Partial *16S rRNA* gene sequencing was therefore used exclusively to confirm the identity of representative isolates rather than to characterize the genetic diversity of circulating strains.

Overall, *L. monocytogenes* was detected in 27.33% (173/633) of raw milk samples collected from dairy farms in Pichincha and Manabí provinces. Positive samples were identified in small-scale [35.84% (62/173)], medium-scale [36.99% (64/173)], and large-scale producers [27.17% (47/173)], indicating that contamination may occur across different production systems. The moderate agreement observed between qPCR and Bacterial culture (κ ≈ 0.6) reflects the complementary nature of these methods. Culture verifies only living organisms capable of growth, whereas qPCR finds bacterial DNA regardless of cell viability. Consistent with this interpretation, qPCR demonstrated high relative sensitivity (92.35%), specificity (96.54%), positive predictive value (90.75%), and negative predictive value (97.17%), supporting its usefulness as a rapid screening tool while reinforcing the continued importance of Bacterial culture for microbiological confirmation.

The disappearance of the association between producer category and *L. monocytogenes* detection after multivariable adjustment indicates that the crude differences observed among producer categories were likely influenced by the unequal distribution of farms across provinces and climatic seasons. Consequently, producer category did not represent an independent risk factor in the final model.

The significantly higher prevalence of *L. monocytogenes* observed in Pichincha compared with Manabí may be explained by several factors. Although the present study was not designed to identify the specific sources of contamination, differences in dairy production systems, environmental conditions, farm management practices, and hygienic measures during milking could have contributed to the observed regional variation. Pichincha is a province of Ecuador that is renowned for its significant dairy production. It is distinguished by its high concentration of specialized dairy farms and intensive milk production systems. These systems are characterized by increased animal density, frequent movement of animals and equipment, and more frequent handling of bulk milk. This combination of factors facilitate the persistence and dissemination of *L. monocytogenes*. In contrast, dairy production in Manabí is generally more extensive, with different climatic conditions and management practices that may influence the environmental survival and transmission of this pathogen. Environmental reservoirs also play an important role in the epidemiology of *L. monocytogenes*. The bacterium is widely distributed in soil, water, silage, feces, and farm environments, and contamination of raw milk may occur through contact with these sources during milking or subsequent handling. Consequently, regional differences in environmental conditions, feeding practices, hygiene protocols, and farm biosecurity may partially explain the higher prevalence detected in Pichincha. Nevertheless, because these factors were not directly evaluated in the present study, these explanations should be considered hypotheses that warrant further investigation through studies specifically designed to assess farm-level risk factors.

The 16 samples classified as false positives relative to Bacterial culture (qPCR-positive/culture-negative) may have several biological and methodological explanations. Because qPCR detects bacterial DNA rather than viable cells, positive amplification may occur even when bacterial viability has been reduced during sample handling or enrichment. In addition, *L. monocytogenes* may persist in a viable but non-culturable (VBNC) state or be present at very low concentrations, allowing molecular detection but preventing successful isolation by conventional culture methods. Furthermore, selective culture may be affected by bacterial stress or competition with accompanying microbiota, reducing the recovery of viable organisms. Therefore, these results should not necessarily be interpreted as true false-positive molecular reactions but rather as discrepancies between two diagnostic methods with different analytical principles and sensitivities.

Although post-enrichment qPCR may detect DNA from both viable and non-viable bacterial cells, the objective of the present analysis was not to validate qPCR as a measure of bacterial viability but rather to evaluate its relative diagnostic performance compared with the conventional culture method. In accordance with WOAH recommendations, relative sensitivity, relative specificity, PPV, and NPV were calculated to assess the level of agreement between both diagnostic approaches, acknowledging that Bacterial culture is itself an imperfect reference method. Therefore, these parameters should be interpreted as measures of comparative diagnostic performance rather than absolute indicators of diagnostic accuracy.

Compared with conventional culture methods, post-enrichment qPCR offers the advantages of greater analytical sensitivity, faster turnaround time, and high analytical specificity through the amplification of species-specific genetic targets. Previous studies have reported that qPCR assays targeting *L. monocytogenes* are capable of detecting very low bacterial concentrations after enrichment, often corresponding to fewer than 10–100 CFU/mL depending on the enrichment protocol, DNA extraction procedure, and amplification chemistry employed. In contrast, bacterial iso requires the recovery of viable organisms and may fail when bacterial numbers are low or cells are stressed or in a viable but non-culturable (VBNC) state. These methodological differences likely contributed to the slightly higher detection rate observed by post-enrichment qPCR (27.33%) compared with Bacterial culture (26.86%) in the present study. Nevertheless, because DNA extraction was performed after selective enrichment, the present assay should be considered a qualitative detection method rather than a direct measure of the initial bacterial concentration in raw milk.

The occurrence of *L. monocytogenes* in more than one-quarter of the analyzed milk samples demonstrates that contamination of raw bovine milk occurs in the evaluated regions. However, these findings should be interpreted cautiously. Bacterial enumeration was not performed, and molecular quantification was conducted after selective enrichment; therefore, the estimated gene copy numbers do not represent the original concentration of *L. monocytogenes* in raw milk. Instead, they reflect post-enrichment molecular measurements that facilitate pathogen detection but cannot be directly translated into viable bacterial counts or consumer exposure levels. Consequently, the present study documents pathogen occurrence rather than quantitative contamination or microbiological risk.

The likelihood of listeriosis associated with contaminated dairy products depends on multiple factors, including bacterial concentration, consumption frequency, storage conditions, and host susceptibility. Previous studies indicate that healthy individuals generally require relatively high exposure levels (approximately 10^6^ CFU/g or mL) to develop disease, whereas susceptible populations—including pregnant women, older adults, and immunocompromised individuals—may become infected after exposure to substantially lower doses, sometimes as low as 10^2^ CFU [44,45,46,47,48,49]. In the present study, qPCR estimated post-enrichment gene copy numbers ranging from approximately 7.84 × 10^3^ to 2.69 × 10^14^ genome equivalents per milliliter. Likewise, the post-enrichment qPCR quantification presented in this study should not be interpreted as a direct estimate of the bacterial concentration in raw milk. Since DNA extraction was performed after selective enrichment, bacterial multiplication during incubation precludes direct inference of the initial concentration of *L. monocytogenes* in the original samples. Consequently, the estimated gene copy numbers should be regarded only as post-enrichment molecular measurements that facilitate pathogen detection rather than bacterial enumeration. Therefore, these data cannot be used to infer infectious dose or directly estimate consumer exposure or public health risk. 

Potential sources of contamination include inadequate udder hygiene, contaminated water, environmental reservoirs such as soil and feces, insufficient sanitation of milking equipment and storage tanks, and biofilm formation within dairy facilities. Environmental conditions, including humidity, temperature, and the presence of organic matter, may further facilitate bacterial persistence [32,50,51,52]. Nevertheless, these factors were not directly evaluated in the present investigation and should therefore be regarded as plausible explanations supported by previous literature rather than confirmed determinants of contamination in the studied farms.

A major strength of this study is the combined use of molecular and microbiological methods to investigate *L. monocytogenes* occurrence in a relatively large number of raw milk samples collected from two important dairy-producing provinces of Ecuador. However, several limitations should also be acknowledged. Besides the absence of bacterial enumeration, no quantitative microbial risk assessment was performed, preventing direct estimation of consumer exposure. Although only one bulk tank milk sample was collected from each participating farm, unmeasured environmental, management, or regional characteristics shared among farms may have influenced the observed prevalence of *L. monocytogenes*. There was no within-farm clustering pattern that necessitated cluster-based statistical analysis because each farm only provided one observation. These factors should therefore be interpreted as potential sources of residual confounding rather than statistical dependence among observations. These limitations should be considered when interpreting prevalence estimates and extrapolating the results to other dairy production systems.

Statistically significant differences in the prevalence of *L. monocytogenes* were observed between the two provinces evaluated (*p* < 0.0001), with a higher proportion of positive milk samples detected in Pichincha than in Manabí. Multivariable logistic regression analysis further demonstrated that milk samples collected in Pichincha had significantly higher odds of testing positive than those collected in Manabí, whereas samples obtained during the dry season had significantly lower odds of positivity than those collected during the rainy season. In contrast, producer size was not significantly associated with *L. monocytogenes* detection after simultaneously accounting for the effects of province and climatic season in the multivariable model.

The higher occurrence observed in Pichincha may be associated with differences in environmental conditions, altitude, climatic characteristics, production systems, or farm management practices. Likewise, seasonal variation may reflect changes in environmental humidity, temperature, or sanitation conditions that influence bacterial survival and dissemination. However, because these variables were not directly measured in the present study, these explanations should be considered hypothetical and require confirmation through studies specifically designed to evaluate farm-level risk factors. Similarly, although differences among producer categories were observed descriptively, the absence of statistical significance after adjustment suggests that producer size alone may not be an independent determinant of contamination.

These findings emphasize that contamination with *L. monocytogenes* is likely influenced by multiple interacting factors rather than by farm size alone. Consequently, preventive measures should focus on strengthening hygiene practices throughout the production chain, including udder preparation before milking, sanitation of milking equipment, appropriate cleaning of storage tanks, and implementation of standardized monitoring programs based on Good Milking Practices and Hazard Analysis and Critical Control Point (HACCP) principles [53].

The prevalence observed in this study differs from the limited information currently available for raw bovine milk in Ecuador. A previous study conducted in Pichincha reported the detection of *Listeria* spp. in only 1.67% (1/60) of raw milk samples [54]. The substantially higher prevalence identified in the present study may be explained by differences in sampling strategy, sample size, diagnostic methodology, and the higher analytical sensitivity achieved by combining selective enrichment with qPCR confirmation. In addition, temporal variations in farm management practices and environmental conditions may also have contributed to the observed differences [55,56].

Variability in the reported prevalence of *L. monocytogenes* has also been described in studies from other countries, although direct comparisons should be interpreted cautiously because differences in study design, diagnostic methodology, sampling strategy, and host species may substantially influence the reported prevalence [57,58].

Evidence from foodborne outbreaks further demonstrates the importance of maintaining strict microbiological control throughout the dairy production chain. For example, a listeriosis outbreak in Canada during 2015–2016 was associated with post-pasteurization contamination of chocolate milk [59]. Likewise, a recent systematic review and meta-analysis including 173 studies reported an overall prevalence of *L. monocytogenes* of 8.69% in milk and dairy products, including 3.44% in raw milk and 0.60% in pasteurized milk [60]. The same review identified South America among the regions reporting the highest frequencies of positive samples, reinforcing the need for continued surveillance and implementation of preventive measures throughout dairy production.

The ability of *L. monocytogenes* to survive and multiply under refrigeration conditions further increases its significance as a foodborne pathogen, particularly in dairy products characterized by high water activity and near-neutral pH [22,23,33,34,57]. Although the present study did not evaluate bacterial growth during storage, this characteristic highlights the importance of maintaining appropriate cold-chain management and minimizing post-harvest contamination to reduce the likelihood of bacterial persistence in dairy products.

Although antimicrobial resistance was not investigated in the present study, previous research has shown that *L. monocytogenes* may acquire resistance determinants through horizontal gene transfer, biofilm formation, and persistence mechanisms, thereby enhancing its survival in food-processing environments [61]. These adaptive characteristics reinforce the importance of continuous microbiological surveillance and strict hygiene practices throughout dairy production to reduce bacterial persistence and the potential dissemination of resistant strains.

Overall, the findings of this study confirm the occurrence of *L. monocytogenes* in raw bovine milk from two major dairy-producing provinces of Ecuador and provide valuable baseline epidemiological data for a region where information on this pathogen remains limited. The combined use of qPCR and conventional Bacterial culture improved pathogen detection and demonstrated that molecular methods represent a valuable complementary tool for surveillance programs while culture-based methods remain essential for confirming bacterial viability.

Nevertheless, the results should be interpreted within the limitations of the study. First, bacterial enumeration was not performed, and molecular quantification was conducted only after selective enrichment; therefore, the estimated gene copy numbers cannot be interpreted as the original bacterial concentration in raw milk or as viable bacterial counts. Second, no quantitative microbial risk assessment was performed, precluding direct estimation of consumer exposure or public health risk. Third, only five representative isolates were subjected to partial *16S rRNA* gene sequencing, which was intended exclusively to confirm bacterial identity rather than characterize the genetic diversity of circulating strains. Consequently, although the present findings demonstrate the occurrence of *L. monocytogenes* in raw milk, they should not be interpreted as evidence of the magnitude of consumer risk or pathogen dissemination at the population level.

Future studies should incorporate direct bacterial enumeration (CFU/mL), quantitative microbial risk assessment, and farm-level risk factor analysis to better characterize contamination pathways and estimate consumer exposure. In addition, sequencing a larger number of isolates using higher-resolution molecular approaches, such as multilocus sequence typing (MLST) or whole-genome sequencing (WGS), would provide a more comprehensive understanding of the genetic diversity, epidemiology, and circulation of *L. monocytogenes* in Ecuadorian dairy production systems.

A limitation of this study is that only five representative isolates were subjected to partial *16S rRNA* gene sequencing for molecular confirmation. Although these sequences confirmed the identity of representative cultured isolates previously identified by phenotypic methods and qPCR, they were not intended to characterize the genetic diversity of all *L. monocytogenes* isolates recovered in this study. Sequencing a larger number of isolates or applying higher-resolution molecular typing approaches, such as multilocus sequence typing (MLST) or whole-genome sequencing (WGS), would provide a more comprehensive characterization of the genetic diversity, transmission dynamics, and epidemiology of *L. monocytogenes* circulating in Ecuadorian dairy production systems. Another limitation is that PCR inhibition was minimized by DNA dilution rather than being directly monitored using an internal amplification control or endogenous reference gene. Although dilution is a widely used strategy to reduce inhibitory effects in milk samples, the absence of an inhibition control precluded direct quantification of residual PCR inhibition in individual reactions.

## 5. Conclusions

After completing the isolation and identification of *L. monocytogenes* in raw bovine milk samples from Pichincha and Manabí provinces, Ecuador, we found that the prevalence of *L. monocytogenes* was 26.86% by culture and 27.33% by qPCR. The substantial agreement observed between both diagnostic methods demonstrates the reliability of qPCR as a rapid and sensitive complementary tool for pathogen detection. Furthermore, because the molecular analyses were performed after selective enrichment, the estimated gene copy numbers represent post-enrichment molecular measurements and should not be interpreted as the original concentration of *L. monocytogenes* in raw milk. Therefore, these results confirm the occurrence of the pathogen but do not provide a direct quantification of viable bacteria present in the original samples. The detection of *L. monocytogenes* in raw bovine milk highlights the need to strengthen hygienic practices during milking, milk handling, and distribution, as well as to reinforce surveillance programs aimed at reducing contamination throughout the dairy production chain. These findings provide valuable baseline epidemiological information on the occurrence of *L. monocytogenes* in raw milk in Ecuador and contribute to future monitoring and control strategies under a One Health approach. However, future studies should incorporate direct bacterial enumeration methods (e.g., CFU/mL) and quantitative microbial risk assessment to better estimate consumer exposure and the potential implications for public health.

## Figures and Tables

**Table 1:** Primers and hydrolysis probes used.

Primers and Hydrolysis Probes	Target	Sequence (5′–3′)	Amplicon	Reference
hlyQF	*hly* gene	5′-CAT GGC ACC ACC AGC ATC T-3′	106 bp	[39]
hlyQR		5′-ATC CGC GTG TTT CTT TTC GA-3′		
hlyQP		5′-FAM-CGC CTG CAA GTC CTA AGA CGC CA-TAMRA-3′		
27F	*16S*	5′-TGGTAGTCCACGCCCTAAAC-3′	~1465 bp	[40]
1482R		5′-GACGGGCGGTGTGTRCA-3′		

**Table 2:** Phenotypic characterization with qPCR diagnosis of *L. monocytogenes* in relation to the province of origin of the samples, size of the producer and percentage of positivity of the bacteria.

Province	Type of Producer	*L. monocytogenes*
Total positives		26.86% (170/633)
Pichincha	Small	36.21% (42/116)
	Medium	34.48% (40/116)
	Large	29.31% (34/116)
	Total Pichincha	68.24% (116/170) *
Manabí	Small	33.33% (18/54)
	Medium	37.04% (20/54)
	Large	29.63% (16/54)
	Total Manabí	31.76% (54/170)

* = statistical difference between isolation of bacteria in the provinces.

**Table 3:** Multivariable logistic regression model between the presence/absence of *L. monocytogenes* by province, producer size and climatic season.

Variable	Crude OR	95% CI	*p* Value	Adjusted OR	95% CI	*p* Value
Province						
Pichincha vs. Manabí	2.68	1.85–3.88	<0.001	1.74	1.16–2.62	0.008
Climatic season						
Rainy vs. Dry	6.87	4.66–10.13	<0.001	5.95	3.98–8.90	<0.001
Producer category						
Medium vs. Large	1.06	0.68–1.65	0.793	0.91	0.56–1.48	0.698
Small vs. Large	0.84	0.54–1.30	0.430	0.88	0.54–1.43	0.611

**Table 4:** qPCR diagnosis of *L. monocytogenes* in enriched milk, in relation to the province of the samples, size of the producer and percentage of positivity of the bacteria.

Province	Type of Producer	*L. monocytogenes*
Total positives		27.33% (173/633)
Pichincha	Small	37.40% (49/131)
	Medium	36.64% (48/131)
	Large	25.95% (34/131)
	Total Pichincha	75.72% (131/173) *
Manabí	Small	30.95% (13/42)
	Medium	38.10% (16/42)
	Large	30.95% (13/42)
	Total Manabí	24.28% (42/173)

* = statistical difference between isolation of bacteria in the provinces.

**Table 5:** Contingency table comparing post-enrichment qPCR and Bacterial culture for the detection of *L. monocytogenes* in raw bovine milk samples.

Post-Enrichment qPCR	Bacterial Culture (+)	Bacterial Culture (−)	Total
Positive	157 (true positive)	16 (false positive)	173
Negative	13 (false negative)	447 (true negative)	460
Total	170	463	633

## Data Availability

The original contributions presented in this study are included in the article/Supplementary Materials. Further inquiries can be directed to the corresponding author.

## Supplementary Materials

The following supporting information can be downloaded at: https://www.mdpi.com/article/10.3390/vetsci13090871/s1.
